# The Impact of Termiticides on Termite Corpse Management

**DOI:** 10.3390/insects16020208

**Published:** 2025-02-14

**Authors:** Jizhe Shi, Austin Merchant, Xuguo Zhou

**Affiliations:** 1 Department of Entomology, Martin-Gatton College of Agriculture, Food and Environment, University of Kentucky, Lexington, KY 40546, USA; jizhe.shi@syngenta.com (J.S.); a95merch@gmail.com (A.M.); 2 Department of Crop Protection Development, Syngenta (China) Investment Co., Ltd., Shanghai 200131, China; 3 Faculty of Science, University of Toyama, Gofuku, Toyama 930-8555, Japan; 4 Department of Entomology, School of Integrative Biology, College of Liberal Arts & Sciences, University of Illinois Urbana-Champaign, Urbana, IL 61801, USA

**Keywords:** termiticides, undertaking behavior, *Reticulitermes flavipes*, chemical ecology, pest management

## Abstract

Soil treatments and baits are two primary chemical control strategies for subterranean termites. Baiting is targeted and eco-friendly but requires ongoing maintenance, while soil treatments provide immediate, long-lasting protection with potential environmental concerns. Previously, we found that termites differentially manage deceased individuals based on their postmortem chemical signatures, potentially circumventing chemical controls. Given the distinct differences in the synthetic termiticides used for soil treatments (fast-acting) and baits (slow-releasing), we hypothesized that termites would respond differently to corpses treated with these two methods. To test this hypothesis, in *Reticulitermes flavipes*, we (1) profiled postmortem chemicals in termites exposed to different termiticides and (2) documented live termite responses to these corpses. Significant variations in postmortem chemical signatures, particularly 3-octanol and 3-octanone, were found among termites exposed to different termiticides, especially bifenthrin and fipronil. However, these variations did not lead to significantly different undertaking behaviors, indicating a complex relationship between death cues and termite behavior. Contrary to our hypothesis, except for bifenthrin, the fundamental undertaking behaviors were consistent despite differences in retrieval timing. This suggests that termiticides alone do not fully dictate termite undertaking behavior. Understanding termite corpse management is crucial for evaluating termiticide effectiveness, highlighting the need for an integrated pest management approach.

## Simple Summary

This study explores how chemical treatments for controlling termites affect the behavior of surviving termites toward their deceased nestmates. Focusing on the eastern subterranean termite, *Reticulitermes flavipes*, it examines the effects of two primary termite control methods: soil treatments and baits. The overall goal of this research is to clarify how these treatments impact the volatile profile of termite corpses and subsequent behaviors of living termites. Significant differences in volatiles, such as 3-octanol and 3-octanone, were detected in corpses resulting from exposures to the termiticides bifenthrin and fipronil. These chemical changes, however, did not consistently alter behaviors such as corpse removal or cannibalism, suggesting a complex interplay between chemical signals and termite behavior. Apart from bifenthrin, behavioral responses showed no significant differences across treatments. These findings suggest that while postmortem chemicals do influence behavior, they do not solely dictate how termites interact with their dead nestmates. These combined results underscore the importance of understanding termite undertaking behavior to improve pest management strategies, paving the way for more effective and environmentally sustainable control practices.

## 1. Introduction

Over 3100 termite species have been described, of which 183 cause damage to human buildings [1,2,3]. Most of these 183 species are subterranean termites. Termites are serious pests to crops, buildings, and wooden products, causing a significant economic loss of more than USD 2 billion annually across the world [4]. The use of chemical pesticides is the primary management method, and many products are used worldwide to control termites [5,6,7].

Liquid soil treatment, a traditional method of pest control, involves applying liquid termiticides to the soil around and beneath buildings to form a chemical barrier against subterranean termites [8]. Commonly used termiticides include bifenthrin (Baseline^™^, Talstar^®^ Professional), fipronil (Termidor^®^), imidacloprid (Premise^®^), chlorantraniliprole (Altriset^®^), and chlorfenapyr (Phantom^®^), which are noted for their effectiveness in eliminating termites that penetrate treated soil [9]. Products containing these chemicals have fundamentally different effects on termites. Bifenthrin, a pyrethroid, disrupts termite nervous systems, leading to paralysis and death. Its residual impacts also cause termites to spread the chemical to their colonies, leading to colony-wide control. However, bifenthrin is highly repellent, causing termites to avoid treated soil [10,11,12]. Su and colleagues suggest that chemicals emitted from decomposing termite corpses add a secondary layer of repellency [13]. In addition to repellents like bifenthrin, there are also non-repellent liquid soil treatments. For example, fipronil, a phenyl pyrazole insecticide, eliminates termites by obstructing GABA-regulated chloride channels, inducing hyper-excitation in their central nervous systems [14]. Imidacloprid, a neonicotinoid, blocks nicotinic acetylcholine receptors, causing paralysis and death [15]. The use of fipronil and imidacloprid in agriculture is severely restricted in the US and EU due to their acute toxicity to non-target arthropods, especially pollinators like honeybees [16,17]. Their use is now mostly confined to urban pest control and pet products [18,19]. Chlorantraniliprole, part of the anthranilic diamide group, acts on ryanodine receptors in insect muscle fibers, causing paralysis and death over time [20]. Chlorfenapyr, distinct from imidacloprid, fipronil, and pyrethroids, inhibits termites’ energy production. As a pro-insecticide, it transforms into a toxic form that disrupts ATP production, leading to cell dysfunction and death [21].

In contrast to liquid soil treatments, another important strategy for termite control involves the use of baits that combine cellulose with slow-acting, non-repellent insecticides. While liquid soil treatments create immediate and long-lasting chemical barriers that repel or kill termites on contact, bait systems offer a slower, targeted approach by using non-repellent insecticides to eliminate entire colonies, highlighting the trade-off between rapid action and comprehensive colony control. Termiticides used in the bait systems include insect growth regulators that interfere with termite development and slow-acting toxicants [8,22]. Termites consume these baits and distribute them throughout their nest, leading to gradual population decline. One key class of insect growth regulators is chitin synthesis inhibitors, exemplified by hexaflumuron. This compound inhibits ecdysis in major termite genera such as *Reticulitermes* and *Coptotermes*, affecting their ability to shed exuviae during molting, leading to death from cannibalism or dark necrotic lesions [8,23,24]. Hexaflumuron’s delayed toxicity facilitates its spread through a colony by food distribution or cannibalism. Sulfluramid is a notable slow-acting toxin used in termite control that impedes termite energy production by uncoupling oxidative phosphorylation, disrupting the proton gradient within the termite’s body, effectively leading to starvation and death [22,25,26].

Behavioral responses toward dead conspecifics in termites are primarily mediated by chemical death cues [27,28]. Workers can avoid or seal off tunnels leading to areas containing corpses killed by certain termiticides [23,29] or cannibalize them [24]. The repellency of the termiticides themselves and/or death-related chemicals can have an impact on termite behavior [13,30,31,32,33]. How termiticides influence the release of death-related chemicals is still unclear. Termiticides can also possess contact toxicity, which may negatively influence the mobility of termites and in turn impact undertaking behavior [15,29]. It is still uncertain how different causes of death alter the chemical profiles of corpses and whether termites can detect these differences and adapt their behavior accordingly. Understanding how termiticides influence undertaking behavior is essential for better assessing the impacts of insecticides and enhancing their effectiveness in termite control.

In this study, we aimed to investigate how termites respond to nestmates killed by different termiticides. Based on previous studies and our preliminary research, we hypothesized that termites would exhibit different behavioral responses to conspecifics killed by chemicals from liquid soil treatments compared to those from baits. To examine this overarching hypothesis, we integrated ethology with chemical ecology and carried out the following experiments: (1) we profiled the postmortem chemicals in termites exposed to different termiticides, and subsequently (2) documented the behavioral responses of live termites to the treated corpses. The eastern subterranean termite, *Reticulitermes flavipes*, the most widely distributed termite species in North America and an invasive species in Europe [34,35], was used as the model organism in this study. For the termiticides, the seven most extensively used synthetic chemicals in termite control were tested, including bifenthrin, fipronil, imidacloprid, chlorantraniliprole, and chlorfenapyr for liquid soil treatments, as well as hexaflumuron and sulfluramid for bait treatments.

## 2. Materials and Methods

### 2.1. Termite Colony Collection and Maintenance

A total of three *R. flavipes* colonies were used for this study. Two colonies were collected from the Daniel Boone National Forest, Wolfe, KY (R-I: 37°47′38″ N 83°35′55″ W and R-II: 37°47′20″ N 83°35′42″ W), and the third one was obtained from the Arboretum State Botanical Garden of Kentucky at the University of Kentucky, Lexington, KY, USA (A: 38°00′54″ N 84°30′28″ W). The termites were trapped by placing cardboard rolls beneath decomposing wood for a duration of one to two weeks. Once collected, these rolls were transported to the laboratory and the termites were carefully extracted from the rolls. Newly collected termites were initially isolated in Petri dishes lined with moist paper towels for a minimum of 14 days. They were then transferred to plastic containers (dimensions: L: 31.3 cm, W: 23.0 cm, H: 10.2 cm, Pioneer Plastics, Inc., Dixon, KY, USA), which were filled with moist mulch and layered with pinewood boards. This procedure was consistently applied to termites collected from the same tree trunk within a single collection season. The termites were stored in a controlled environment incubator and maintained in constant darkness at 27 ± 1 °C and approximately 90% relative humidity within the boxes. These termites were utilized for experiments within six months of their collection.

### 2.2. Corpse Preparation

Seven termiticides were selected from the two primary termite control strategies, including bifenthrin, fipronil, imidacloprid, chlorantraniliprole, and chlorfenapyr for liquid soil treatments, as well as hexaflumuron and sulfluramid for bait treatments (Table 1). Chemicals were chosen based on their categories and modes of action (Table 1). All termiticides used were of technical grade: bifenthrin (≥98.0%), fipronil (≥95.0%), imidacloprid (≥98.0%), chlorfenapyr (≥98.0%), chlorantraniliprole (≥95.0%), hexaflumuron (≥98.0%), and sulfluramid (≥98.0%) (Sigma-Aldrich, St. Louis, MO, USA). Distilled acetone served as the solvent for all chemicals.

For the liquid soil treatment termiticides, we conducted a preliminary assay to determine the optimal dose and treatment time. A range of acetone solution concentrations was tested for bifenthrin (0.00002%, 0.0002%, 0.002% *w*/*w*), fipronil (0.001%, 0.01%, 0.1% *w*/*w*), imidacloprid (0.004%, 0.02%, 0.1%, 0.5% *w*/*w*), chlorantraniliprole (0.0002%, 0.0004%, 0.0006% *w*/*w*), and chlorfenapyr (0.004%, 0.006%, 0.008% *w*/*w*). A 0.5 μL dose of each chemical’s acetone solution was topically applied to the pronotums of termite workers using a repeating dispenser and syringe. The treated termites were then placed in a Petri dish lined with a moistened paper towel and incubated at 27 ± 1 °C. Mortality was monitored hourly, leading to the selection of the following specific treatment concentrations and times for each termiticide based on the highest observed mortality rates: 0.0002% bifenthrin at 5 h, 0.01% fipronil at 8 h, 0.5% imidacloprid at 16 h, 0.0006% chlorantraniliprole at 3 days, and 0.006% chlorfenapyr at 24 h. The control groups were treated with an equivalent volume of acetone. For the bait termiticides, hexaflumuron and sulfluramid, a dietary application method was utilized. The treatment dosages and durations were informed by previous research [22,36]. Paper disks with a 3.5 cm diameter, made from unbleached paper towels, were treated with 200 μL of either 0.01% *w*/*w* hexaflumuron or 0.003% *w*/*w* sulfluramid acetone solution and air-dried in a fume hood for 40 min. Fifty workers were then placed in Petri dishes lined with four layers of these treated paper disks, along with 200 μL of distilled water. The treated termites were kept in an incubator set at 27 ± 1 °C with ~90% relative humidity in continuous darkness for predetermined durations (21 days for hexaflumuron and 25 days for sulfluramid). Control groups received acetone-treated disks only. Termite mortality was defined as the absence of coordinated movement upon stimulation with a fine brush. The latency to mortality varied depending on the termiticide employed. To establish precise postmortem intervals, corpses were collected using different protocols tailored to the action time of each termiticide.

For the assessment of acute termiticides, corpses treated with bifenthrin, fipronil, imidacloprid, and chlorfenapyr were collected on an hourly basis, starting immediately after treatment and lasting for 5, 8, 16, and 24 h, respectively. In contrast, for termiticides with a delayed mode of action, such as chlorantraniliprole, hexaflumuron, and sulfluramid, which required more than 24 h to induce mortality, corpses were initially removed after the entire treatment duration. Post-treatment, corpses were then collected at 2 h intervals during a 12 h window each day across three consecutive days. This approach was optimized based on preliminary studies to yield the highest number of corpses for subsequent analyses. The commencement of collection was designated as the 0 h postmortem time for each termiticide treatment. Once collected, each corpse was placed in an unsealed 3.5 cm Petri dish and stored in an incubator set to 27 ± 1 °C with approximately 90% relative humidity under constant darkness to simulate environmental conditions conducive to postmortem chemical changes. Thus, to effectively capture the dynamics of death-related chemical release, postmortem time points were set at 0, 1, 2, 4, 8, 16, 32, and 64 h for the behavioral and chemical assays, based on prior research [37].

### 2.3. Profiling Postmortem Chemicals of Corpses Killed by Different Termiticides

To characterize the chemical profile associated with termite mortality, corpses resulting from different termiticides were sampled at designated postmortem intervals (0, 1, 2, 4, 8, 16, 32, and 64 h). Chemical profiling was performed using an Agilent Technologies gas chromatograph equipped with a 30 m × 0.25 mm × 0.25 µm DB-5 capillary column and helium as the carrier gas. The GC temperature program commenced at 40 °C with a 2 min hold, followed by a ramp to 320 °C at a rate of 10 °C/min. The injector temperature was maintained at 280 °C. Compound identification was facilitated by coupling the GC with an Agilent Technologies 5975 mass spectrometer (MS) and referencing the NIST/NIH/EPA Mass Spectral Library.

The detection and quantification of death-related volatiles (3-octanol, 3-octanone, phenol, and indole) were conducted using Solid Phase Microextraction/Gas Chromatography–Mass Spectrometry (SPME/GC-MS) and hexane extraction/GC-MS, respectively. Three colonies were selected for each termiticide, with three technical replicates per colony. For SPME, a 100 µm Polydimethylsiloxane (PDMS) SPME fiber (Agilent Technologies, Santa Clara, CA, USA) was introduced into a 2 mL glass vial containing 15 termite corpses. The vial was sealed with an 11 mm PTFE/red rubber septa crimp cap (Agilent Technologies, Santa Clara, CA, USA). The fiber was exposed to the vial’s headspace above the corpses without direct contact for 15 min to adsorb the volatiles. Subsequently, the fiber was injected into the GC inlet for 1 min for GC-MS analysis. Hexane extraction was employed to quantify the volatiles initially identified by SPME. To this end, 200 µL of distilled hexane containing a 19-carbon hydrocarbon (n-nonadecane, 10 ng/µL) as an internal standard was added to the vials with 15 corpses. After a 10 min extraction period, 2 µL of the hexane solution was taken for GC-MS analysis.

To track the temporal variation in oleic acid, indicative of the accumulation of death-related fatty acids as worker corpses aged, a separate procedure was utilized. Ten corpses were subjected to a 10 min extraction in 300 µL of distilled hexane with n-nonadecane serving as an internal standard at a concentration of 10 ng/µL. The resulting supernatant was transferred to a fresh vial, to which 200 µL of 10% *w*/*w* BF3-methanol was added, and the mixture was then reacted for 10 min at 60 °C. A 2 µL sample of the reaction mixture was analyzed by the GC to quantify oleic acid alongside the related methyl ester (methyl cis-9-octadecenoate).

### 2.4. Behavioral Assay with Corpses Treated with Different Termiticides at Different Postmortem Times

**Figure 1:**
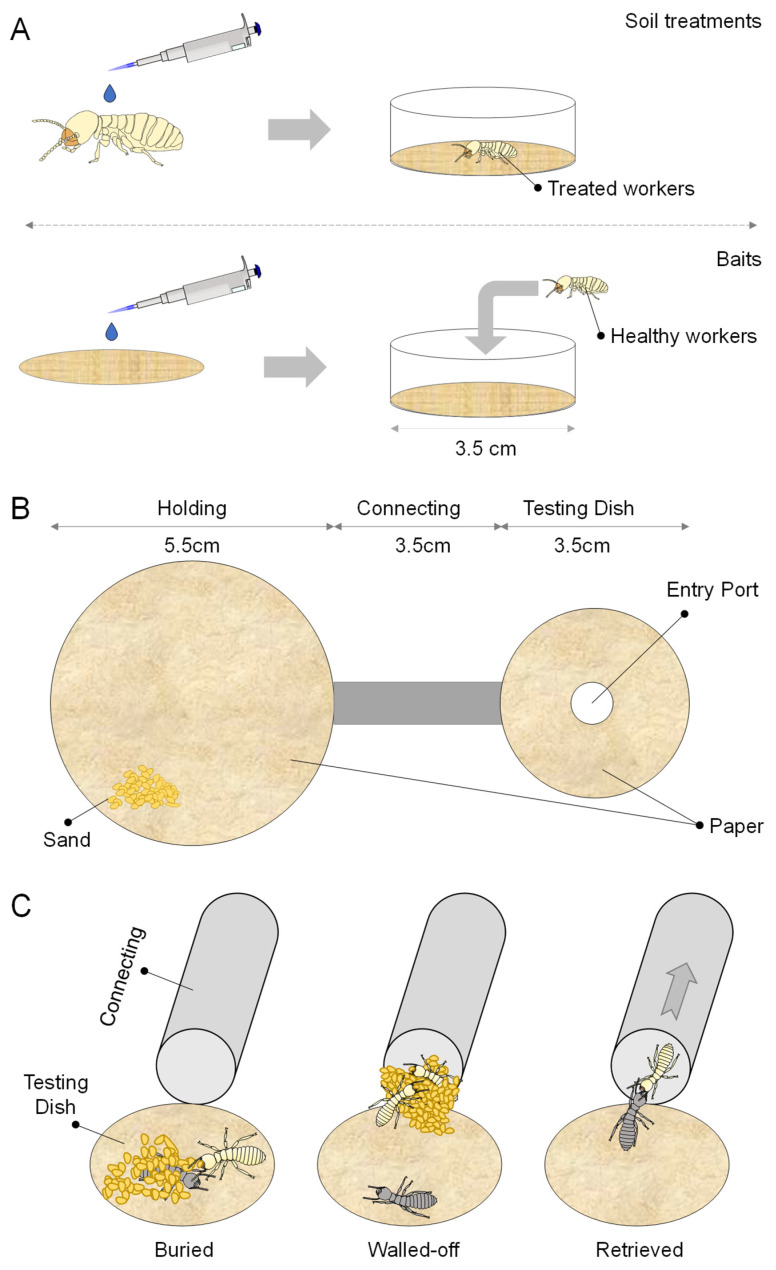
Experimental design. (**A**) Illustration of termiticide treatments, including topical application of liquid soil termiticides, and dietary exposure to termite baits; (**B**) schematic drawing of experimental setup for subsequent behavioral assay; and (**C**) behavioral responses and phenotypes in corpse management, including burial, walling off, retrieval, and eventual cannibalism.

To assess the undertaking responses, corpses killed with different termiticides at various postmortem times (0, 1, 2, 4, 8, 16, 32, and 64 h) were introduced into a two-dish assay system (Figure 1). The setup consisted of a 5.5 cm Petri dish serving as a living chamber, connected to a 3.5 cm Petri dish, used as a testing chamber, by a 3.5 cm long plastic tube with a diameter of 7 mm. Both chambers were covered with unbleached paper towels moistened with 200 μL of distilled water. Additionally, 1 g of sand dampened with 150 μL of distilled water was provided in the living chamber as burial material for use in the undertaking process.

Twenty-nine workers and one soldier were placed into the living chamber, where they were allowed to acclimate for three days prior to the initiation of the assay. For each setup, a single corpse was introduced into the testing chamber through a 0.5 cm aperture in the chamber lid. The termites’ behavioral responses to each corpse were documented over a 24 h period using a Canon VIXIA HF G10 camcorder. The disposition of the corpse was categorized as retrieval, burial, or wall-off. A burial response was identified when sand was deposited onto the corpse. A wall-off response was noted when sand particles accumulated at the testing chamber’s entrance. Retrieval was inferred when a corpse was moved back to the living chamber for cannibalism through the plastic tubing connecting the two chambers. The retrieval time was recorded when a corpse was first moved to the entrance of the living chamber. Each of the 3 termite colonies were subjected to 10 replicates of the assay.

### 2.5. Data Analysis

Statistical analyses were performed utilizing JMP 14.0 software (SAS Institute Inc., Cary, NC, USA). Behavioral responses associated with termite corpses—specifically dispositional responses and retrieval times—as well as the amounts of death-associated chemicals (early death cues: 3-octanol and 3-octanone; late death volatiles: phenol and indole; and a fatty acid representative: oleic acid) were analyzed for variance among different termiticides and across various postmortem intervals. Behavioral responses were classified into three categories: retrieval, burial, and wall-off. The Shapiro–Wilk W test was employed to assess the normality of the continuous variables, which included the retrieval time and the concentrations of death-related chemicals. To satisfy the prerequisites of parametric testing procedures, the chemical data were log-transformed. An ANOVA was conducted to determine the effects of the termiticides, the postmortem times, and their interaction on the measured chemical quantities and termite behaviors. This was followed by post hoc pairwise comparison using the Tukey–Kramer HSD test to elucidate specific differences. Graphical representations of the data were created with Prism 9 for macOS (GraphPad Prism 9.2.0, GraphPad Software, San Diego, CA, USA), facilitating the visualization of patterns and results within our findings.

## 3. Results

### 3.1. Postmortem Chemical Profiles of Corpses Killed by Different Termiticides

**Figure 2:**
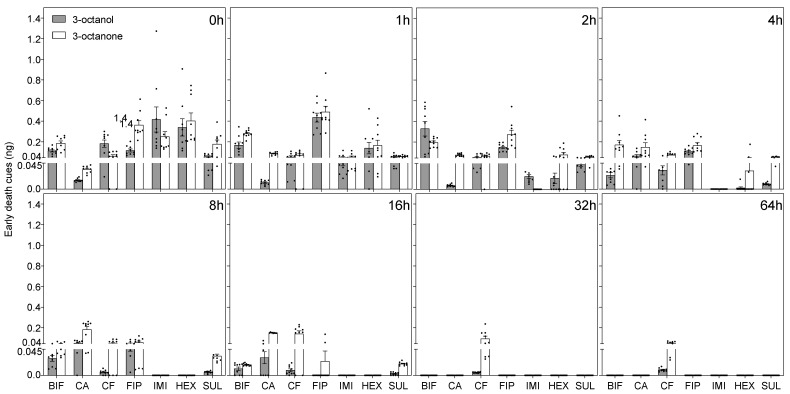
The temporal variation in the early death cues released in *R. flavipes* exposed to different termiticides. Data are shown for two early death cues, 3-octanol and 3-octanone. The mean quantities ± the SEM are plotted over various postmortem time points. 3-Octanone is represented in light gray, and 3-octanol in white. Each group’s sample distribution is shown by unfilled circles, with the different squared sections indicating distinct postmortem time points. The *x*-axis abbreviations represent different termiticide treatments, including BIF (bifenthrin), CA (chlorantraniliprole), CF (chlorfenapyr), FIP (fipronil), IMI (imidacloprid), HEX (hexaflumuron), and SUL (sulfluramid). The data were collected from three colonies, with three replicates per colony.

**Figure 3:**
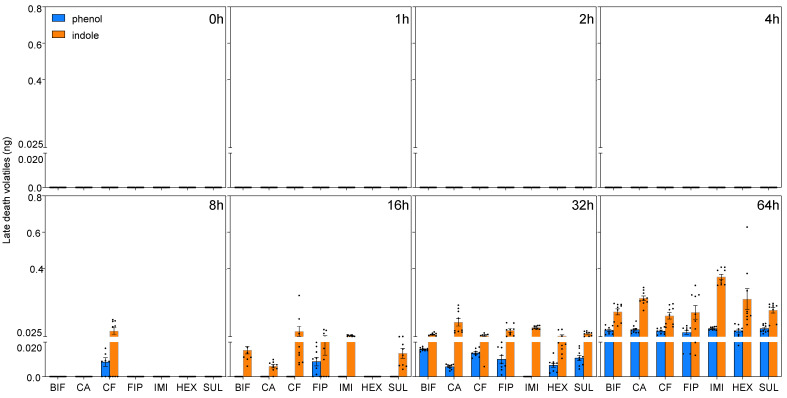
The temporal dynamics of late death volatiles in *R. flavipes* exposed to different termiticides. Data are shown for two late death cues, phenol and indole. The mean quantities ± the SEM are displayed for various postmortem time points. The volatiles are color-coded for clarity (phenol in orange, indole in blue). The sample distributions within each group are indicated by unfilled circles, and the different square sections represent distinct postmortem time points. The *x*-axis abbreviations represent different termiticide treatments, including BIF (bifenthrin), CA (chlorantraniliprole), CF (chlorfenapyr), FIP (fipronil), IMI (imidacloprid), HEX (hexaflumuron), and SUL (sulfluramid). Data compilation involved three colonies, with three replicates each.

**Figure 4:**
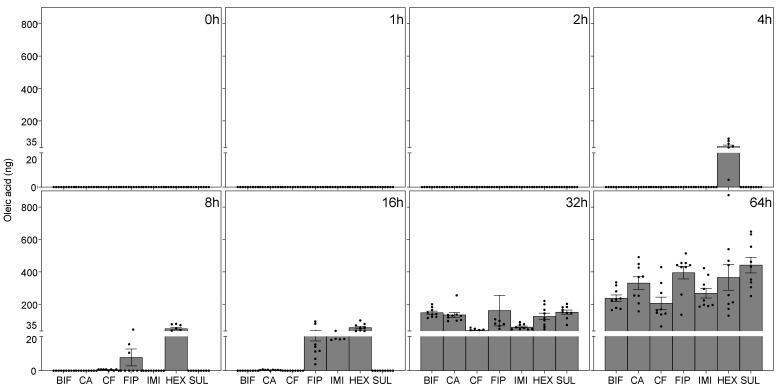
The temporal dynamics of oleic acid in *R. flavipes* exposed to different termiticides. The amount of oleic acid was quantified through hexane extraction, and the results are shown as means + the SEM, with sample distribution represented by black dots. The different squared sections within the panel indicate various postmortem time points. The *x*-axis abbreviations represent different termiticide treatments, including BIF (bifenthrin), CA (chlorantraniliprole), CF (chlorfenapyr), FIP (fipronil), IMI (imidacloprid), HEX (hexaflumuron), and SUL (sulfluramid). The data were collected from three distinct colonies, with three replications.

Chemical analyses revealed significant variations in the quantities and release patterns of death-related chemicals among corpses killed by different termiticides (Figure 2, Figure 3 and Figure 4). Early death cues were detected immediately following death and exhibited an increase, reached peak concentrations, and then subsequently diminished within a span of 16 h in most cases (Figure 2). Analysis of variance revealed significant differences in the total release amounts of 3-octanol and 3-octanone across different termiticides (3-octanol: F(6, 503) = 4.84, *p* < 0.001, *η*^2^ = 0.06; 3-octanone: F(6, 503) = 9.58, *p* < 0.001, *η*^2^ = 0.10). Pairwise comparisons indicated that workers killed by fipronil released significantly higher amounts of 3-octanol compared to those killed by chlorantraniliprole, chlorfenapyr, and sulfluramid, while those killed by bifenthrin released significantly more 3-octanol than those killed by chlorantraniliprole and sulfluramid (*p* < 0.05; Cohen’s d > 0.5). All other pairwise comparisons showed no significant differences (*p* > 0.05; Cohen’s d < 0.5). Regarding 3-octanone, workers killed by fipronil and bifenthrin released significantly greater amounts than those killed by sulfluramid and imidacloprid (*p* < 0.05; Cohen’s d > 0.5), with no significant difference observed between fipronil and bifenthrin (*p* > 0.05; Cohen’s d = 0.49). Workers killed by fipronil exhibited a significantly higher release of 3-octanone compared to those killed by chlorantraniliprole, hexaflumuron, and chlorfenapyr (*p* < 0.05, Cohen’s d = 0.71, 0.72, and 0.79, respectively), whereas those killed by bifenthrin did not (*p* > 0.05; Cohen’s d = 0.22, 0.23, and 0.30, respectively). The release patterns of early death cues varied across workers killed by different termiticides, with those killed by fipronil, bifenthrin, hexaflumuron, and imidacloprid showing rapid release and depletion, peaking within 2 h after death. In contrast, workers killed by chlorantraniliprole, chlorfenapyr, and sulfluramid exhibited a gradual release of early death cues, with relatively lower levels released. Analysis of variance indicated a significant effect of termiticide on the release of 3-octanol (F(7, 347) = 29.52, *p* < 0.001, *η*^2^ = 0.38), with no significant colony effect (F(2, 347) = 0.08, *p* = 0.93, *η*^2^ = 0.0003). For 3-octanone, the effects of both termiticide and colony were significant (termiticide: F(7, 347) = 32.39, *p* < 0.001, *η*^2^ = 0.40; colony: F(2, 347) = 3.13, *p* = 0.04, *η*^2^ = 0.01).

The total released amounts of late death cues, which included phenol, indole, and oleic acid, did not show significant differences across termiticides (phenol: F(6, 503) = 0.09, *p* = 0.99, *η*^2^ = 0.001; indole: F(6, 503) = 1.34, *p* = 0.23, *η*^2^ = 0.02; oleic acid: F(6, 503) = 1.55, *p* = 0.16, *η*^2^ = 0.02) (Figure 3 and Figure 4). Notably, a significant increase in late death cues was typically detected at 32 h postmortem. Late death volatiles, phenol and indole, were not detectable until 16 h postmortem in corpses treated with most termiticides. Similarly, oleic acid was not detectable until 16 h postmortem in corpses treated with bifenthrin, chlorantraniliprole, chlorfenapyr, and sulfluramid, whereas it was detected at 8 h postmortem in fipronil-treated corpses and at 4 h postmortem in hexaflumuron-treated corpses. No significant differences were found in the quantities of phenol and indole among workers killed by different termiticides (phenol: F(20, 129) = 1.24, *p* = 0.25, *η*^2^ = 0.15; indole: F(20, 179) = 0.99, *p* = 0.48, *η*^2^ = 0.05). Different termiticides had a significant effect on the quantity of oleic acid released (F(7, 215) = 9.50, *p* < 0.001, *η*^2^ = 0.26), with no colony effect (F(2, 215) = 0.03, *p* = 0.97, *η*^2^ = 0.0002). No interactions were detected between the effects of termiticide and colony on the quantities of all the death-related chemicals (*p* > 0.05; phenol: *η*^2^ = 0.04; indole: *η*^2^ = 0.04; oleic: *η*^2^ = 0.006; 3-octanol: *η*^2^ = 0.01; 3-octanone: *η*^2^ = 0.01).

### 3.2. Behavioral Responses Towards Corpses Killed by Different Termiticides

**Figure 5:**
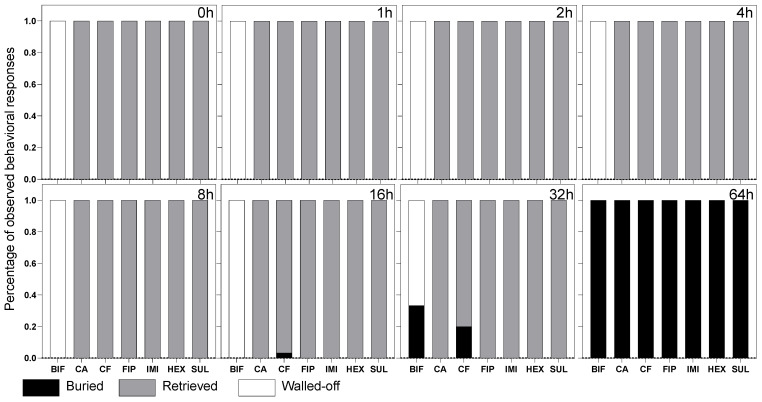
The behavioral responses of *R. flavipes* workers to corpses treated with different termiticides. The different squared sections on the panel represent distinct time points. The behaviors are color-coded for clarity: burial behavior in black, retrieval in gray, and wall-off in white. The *x*-axis abbreviations represent different termiticide treatments, including BIF (bifenthrin), CA (chlorantraniliprole), CF (chlorfenapyr), FIP (fipronil), IMI (imidacloprid), HEX (hexaflumuron), and SUL (sulfluramid). The data presented are an aggregation from three colonies, each subjected to ten replications.

Corpses at ≤32 h postmortem were predominantly transported back to the living chamber by workers, whereas those at 64 h postmortem were always buried on-site. However, response patterns were variable across the different termiticides (Figure 5). Notably, bifenthrin induced a pronounced aversion effect, evidenced by a significant number of wall-off responses (*n* = 200), which were substantially higher compared to the occurrences of burial (*n* = 25) and in stark contrast to the absence of retrieval. Conversely, corpses treated with chlorantraniliprole, chlorfenapyr, sulfluramid, fipronil, imidacloprid, and hexaflumuron predominantly elicited retrieval responses within the first 32 h postmortem. Aside from bifenthrin, other termiticides did not significantly deter termite workers from engaging in retrieval behavior. The burial behavior exhibited variability; corpses treated with chlorfenapyr began to be buried at 16 h postmortem, while those treated with bifenthrin were not buried until 32 h postmortem. Corpses treated with other termiticides were buried only after substantial decomposition, typically observed at 64 h postmortem.

**Figure 6:**
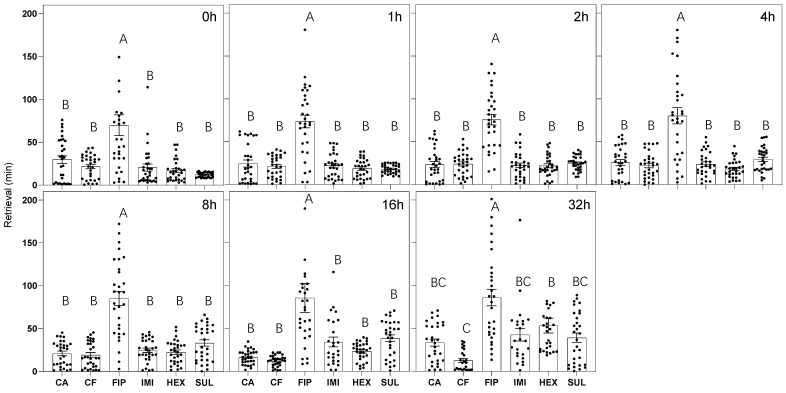
The retrieval times of *R. flavipes* corpses resulting from exposure to different termiticides. The black dots represent individual samples within each group. The *x*-axis abbreviations represent different termiticide treatments, including CA (chlorantraniliprole), CF (chlorfenapyr), FIP (fipronil), IMI (imidacloprid), HEX (hexaflumuron), and SUL (sulfluramid). Data compilation involved three colonies, with ten replications per colony. Groups labeled with identical letters signify no significant difference between these groups.

The retrieval of corpses for cannibalism, a process where workers transported corpses from the testing chamber to the living chamber, varied significantly in timing (Figure 6). Our analysis indicates that both the type of termiticide and the postmortem interval significantly affected retrieval times (termiticide: F(5, 1237) = 126.50, *p* < 0.001, *η*^2^ = 0.33; postmortem time: F(6, 1237) = 5.49, *p* < 0.001, *η*^2^ = 0.02), with a notable interaction effect (F(30, 1237) = 1.77, *p* < 0.01, *η*^2^ = 0.03), suggesting that both the termiticide used and the postmortem time jointly influence the occurrence of retrieval behavior. In the case of fipronil-treated corpses, retrieval times were longer compared to those killed by other termiticides, indicating a possible deterrent effect of fipronil on termite workers. Specifically, corpses treated with fipronil exhibited longer retrieval times compared to those treated with other termiticides across all postmortem intervals (*p* < 0.001; Cohen’s d > 0.84). For corpses at 0–16 h postmortem, there was no significant difference in retrieval time across termiticides except for fipronil (*p* > 0.05; Cohen’s d < 0.5). At 32 h postmortem, workers killed by hexaflumuron showed significantly longer retrieval time compared to those killed by chlorfenapyr (*p* < 0.01, Cohen’s d = 1.02), although neither of these two showed significant differences in retrieval time compared to those killed with imidacloprid, sulfluramid, or chlorantraniliprole. No effect of colony was detected in our study (F(2, 1237) = 1.67, *p* = 0.19, *η*^2^ = 0.002). Bifenthrin was not included in this analysis given that it did not elicit retrieval behavior in our observations.

## 4. Discussion

### 4.1. Different Release Patterns of Early Death Cues Induced by Termiticides

Social insects, such as termites, exhibit complex behaviors in response to death cues, that is, chemicals released from deceased conspecifics [28,38,39]. Our findings reveal variations in the release of early and late death cues in termite corpses subjected to different termiticide treatments. The corpses of termites that died from exposure to fipronil and bifenthrin treatments released significantly higher levels of death cues at different postmortem times compared to those treated with other termiticides. The release patterns of the early death cues, 3-octanol and 3-octanone, were also influenced by the type of termiticide used. Fipronil and bifenthrin induced a rapid peak and subsequent depletion within 2 h postmortem. Conversely, chlorantraniliprole, chlorfenapyr, and sulfluramid showed a more gradual release. However, the release of late death cues, phenol, indole, and oleic acid, was consistent across the dead individuals resulting from exposure to different termiticides.

Termite workers use a sophisticated chemical communication system to detect and respond to dead nestmates [37]. After the death of an *R. flavipes* worker, two C8 volatiles, 3-octanol and 3-octanone, are released, peak, and then diminish over the next 16 h, regardless of the cause of death. Fatty acids are accumulated during decomposition, and eventually lead to the release of the late death cues, including phenol and indole, at approximately 32 h postmortem. Chemical profiling revealed differences in the release of death-related chemicals, both quantitatively and qualitatively, across deceased workers treated with different termiticides. These patterns are consistent with those observed in corpses killed by freezing [37].

The presence of 3-octanol and 3-octanone in termites is relatively rare and has been primarily documented in *Reticulitermes* species [37,40]. These compounds have been identified in the whole-body extracts of *R. lucifugus*, *R. grassei*, and *R. banyulensis*, alongside *R. flavipes*, which was initially identified as *R. santonensis* [41]. These compounds are also known in ants as alarm pheromones, and include 3-octanone in *Manica mutica* and *M. bradleyi* [42] and both 3-octanol and 3-octanone in *Crematogaster* [43] and *Camponotus* ants [44]. Additionally, 3-octanol has been found in the honeydew of the sooty beech scale, *Ultracoelostoma* spp., with a suggested function of attracting native birds and other insects [45]. These compounds are also prevalent in plants and fungi and are known to contribute to flavor, chemical defense, and fungal growth inhibition [46,47,48,49]. In plants and fungi, fatty acids, specifically linoleic acid, are suggested to be precursors in the biosynthesis of C8 volatiles in an enzyme-catalyzed process [46,48]. In our preliminary study, 3-octanol and 3-octanone were detected in injured *R. flavipes* workers, which suggested that synthesis might happen before death. Although it has long been established that chemicals created during decomposition play a significant role in corpse management in eusocial insects [39], the exact synthesis and release mechanisms of 3-octanol and 3-octanone during death in *R. flavipes*, and the impact of termiticides on this process, remain unclear.

### 4.2. Aversive Effect of Bifenthrin and Fipronil Treatment on Undertaking Behavior

Subterranean termites, including *R. flavipes*, inhabit enclosed nesting structures [28,39,50]. The release of volatile early death cues, specifically 3-octanol and 3-octanone, aids in locating recently deceased individuals [37]. Our findings revealed a higher release of these compounds in bifenthrin- and fipronil-exposed corpses. However, contrary to expectations, this did not result in accelerated retrieval. In cases of bifenthrin exposure, corpses were walled off rather than retrieved for cannibalism, even when freshly killed (within 32 h postmortem). On the other hand, corpses killed by fipronil were eventually retrieved, like those killed by other chemicals, but with a notable delay. This suggests a distinct aversion to *R. flavipes* to corpses treated with bifenthrin and fipronil, particularly during the early postmortem stages.

Pesticides used for termite control are categorized into three types based on their behavioral responses [13]: Type I induces tunnel sealing by termites, Type II results in quick kills leading to tunnel blockage due to dead accumulation, and Type III does not repel termites, allowing them to enter treated areas. Bifenthrin, as part of our study, is known for its repellent properties against termites as in Type I [12]. Our behavioral assays confirmed that bifenthrin-exposed corpses were consistently avoided, leading to wall-off behavior, likely to protect healthy nestmates from hazardous corpses. In contrast, there were no previous reports of the repellency of the other chemicals used in our study [31,51]. This was reflected in our observations, where non-bifenthrin and non-fipronil corpses were generally retrieved and cannibalized at less than 64 h postmortem.

The aversion to certain termiticides may also stem from behavioral resistance, as described in Type II termiticides. Unlike conventional insecticide resistance, which involves genetic and physiological adaptations that reduce or circumvent intoxication upon contact or ingestion, behavioral resistance refers to evolutionary changes in behavior that allow an insect to evade or counteract management tactics. By modifying their behaviors, insects can reduce the effectiveness of pesticides, presenting a distinct challenge in integrated pest management [52]. For instance, the German cockroach, *Blattella germanica*, avoids feeding on sugar baits containing toxicants due to a change in the glucose receptor [53,54]. The horizontal transfer of fipronil can poison the healthy nestmates and immobilize them. During this period, the termites remain alive but are unable to move, which may assist in explaining the delay in corpse retrieval. The immobilization and delay of undertaking freshly fipronil-killed corpses might result in the accumulation of corpses in treated areas. In termites, behavioral resistance has also been observed for fipronil treatment in other species. In *C. formosanus*, the accumulation of dead and decayed corpses in areas treated with fipronil can prevent other nestmates from entering these areas and reduce the efficacy of the termiticide [29]. In *C. formosanus*, with ideal concentrations, fipronil treatment is less effective over 5 m away from the treated area [29], whereas in *R. flavipes*, this distance has been suggested to be over 10 m [55].

Based on the results from this study, resistance to fipronil in subterranean termites is more behavioral than physiological. In our study, fipronil-killed corpses were dragged back toward the colony (away from the treatment site) and subsequently cannibalized, leading to the immobilization of nestmates, suggesting an explanation for this extension of the effective area of fipronil. By using C14-labeled fipronil, horizontal transfer was suggested through body contact, not trophallaxis [56], with nearly 50% of fipronil transferred from exposed termites to other nestmates [57]. This dilution during horizontal transfer would partially explain the recorded effective distance. Other compounds like imidacloprid and chlorantraniliprole have also been reported to significantly decrease termite mobility, affecting their natural behaviors [30,58,59]. However, in our results, no significant differences were detected in undertaking behavior among corpses treated with these chemicals. Imidacloprid has shown to be effective in deterring termite colonization in treated areas [60]. In our study, the average time for corpse retrieval was longer than that reported in previous studies [37], suggesting a delay due to the chosen chemicals. This implies that the chemical nature of termiticides influences the behavioral responses of healthy termites, particularly in their corpse management practices.

### 4.3. Comparison of Liquid Soil Treatments and Baits

In the treatment of subterranean termites, liquid soil treatments and baits represent the two primary strategies, each requiring distinct properties in termiticides. Liquid soil treatments necessitate termiticides that act quickly to either kill or repel termites, thereby preventing them from penetrating the soil and causing structural damage. In contrast, baits are designed to have a slower effect, allowing foraging termites to carry the chemicals back to the colony, facilitating their spread among nest members. While liquid soil treatments aim to affect only a segment of the termite population to prevent soil penetration, baits are intended to impact the entire colony, ultimately leading to its elimination [61].

Contrary to our initial hypothesis, the variation in chemical release patterns during the decomposition of corpses killed by selected termiticides did not result in markedly different behavioral responses. This observation held true across chemicals used in both liquid soil treatments (fipronil, imidacloprid, chlorantraniliprole, and chlorfenapyr) and baits (hexaflumuron and sulfluramid), with the notable exception of bifenthrin. One possible explanation is that while fipronil, imidacloprid, chlorantraniliprole, and chlorfenapyr alter the chemical profiles of termite corpses, these changes do not drastically affect the chemical patterns that trigger specific behaviors. Hexaflumuron and sulfluramid, used in baits, are designed to be slow-acting and interfere with termite development or energy production, but they do not significantly alter the chemical signals in a way that would change termite behavior towards corpses. Another possible explanation is that termite behavioral patterns are influenced not only by chemical signals but also by other factors such as environmental context, social structure, and evolutionary adaptations. These behaviors are robust and not easily altered by changes in chemical signals alone, especially if those changes are not drastic. This comparison highlights that both liquid soil treatments and baits, despite their different modes of action and application methods, do not significantly alter termite undertaking behavior. Understanding this behavior is vital, as it contributes to the overall effectiveness of termite control strategies, particularly in terms of colony management and potential elimination.

The undertaking behavior in response to termite nestmates killed by different termiticides raises an important consideration: the likelihood of the nestmates retrieving and consuming the dead termites, which could lead to horizontal chemical transfer throughout the colony. This undertaking behavior offers a critical perspective for evaluating the efficacy of chemicals used in future termiticides, underscoring the complexity of termite social dynamics and the need for integrated pest management strategies.

## 5. Summary and Perspectives

Soil-treating fast-acting pesticides and slow-releasing baits are the two major strategies used in termite control. To better understand how termites respond to nestmates killed by synthetic compounds, we examined the effects of different termiticides on the postmortem chemical profiles of termite corpses and the subsequent undertaking behavior of surviving termites. We have discovered that the release of early death cues, such as 3-octanol and 3-octanone, varies significantly in termite corpses treated with different termiticides. Notably, corpses exposed to bifenthrin and fipronil exhibited an elevated release of these compounds. However, this did not necessarily translate into accelerated corpse retrieval or cannibalism behaviors, indicating a nuanced relationship between chemical cues and the subsequent termite behavioral responses. Interestingly, except for in bifenthrin-treated corpses, while there were differences in the timing and extent of retrieval and burial behaviors, the fundamental undertaking behaviors remained consistent across all treatments, which challenged our initial hypothesis. This suggests that the chemical nature of a termiticide alone does not solely dictate the termite behaviors, particularly in their response to dead nestmates. The behavioral aspect, especially the potential for horizontal chemical transfer via undertaking behavior, emerges as a critical factor in evaluating the effectiveness of future termiticides. Overall, our research highlights the critical need to consider both chemical properties and behavioral responses when designing and implementing chemical control strategies. These findings have significant implications for termite management in the field. A better understanding of workers’ responses to nestmates killed by termiticides can inform the strategic application of these chemicals. The rapid burial of decomposed corpses suggests that termites can isolate contaminated individuals, potentially limiting the spread of insecticidal compounds within the colony. Targeting this behavior can facilitate colony-wide exposure, improving the efficacy of termite controls. Furthermore, differences in repellency and non-repellency among termiticides highlight the need for tailored/targeted approaches, depending on whether the goal is immediate deterrence or gradual colony elimination.

## Figures and Tables

**Table 1:** Termiticides tested in this study.

Active Ingredient *	Termiticide Type		Chemical Class	Mode of Action **		Treatment	Concentrations (% *w*/*w*)	Corpse Collection
BIF	Liquid soil treatment	Repellent	pyrethroid	3A	Sodium channel modulators	contact	0.0002	5 h
FIP		Non- repellent	phenylpyrazole	2B	GABA-gated chloride channel antagonists	contact	0.01	8 h
IMI			neonicotinoid	4A	Nicotinic acetylcholine receptor (nAChR) competitive modulators	contact	0.5	16 h
CA			anthranilic diamide	28	Ryanodine receptor modulators	contact	0.0006	3 days
CF			pyrrole	13	Uncouplers of oxidative phosphorylation via disruption of proton gradient	contact	0.006	1 day
HEX	Bait		insect growth regulators	15	Inhibitors of chitin biosynthesis affecting CHS1	feeding	0.01	21 days
SUL			slow-acting metabolic inhibitors	13	Same as CF	feeding	0.003	25 days

“*”: Active ingredients involved in termiticide treatments, including bifenthrin (BIF), chlorantraniliprole (CA), chlorfenapyr (CF), fipronil (FIP), imidacloprid (IMI), hexaflumuron (HEX), and sulfluramid (SUL). “**”: Mode of action of active ingredients in termiticide treatments as defined by Insecticide Resistance Action Committee.

## Data Availability

The data can be made available upon request.
